# Effect of Mineral Pitch on the Proliferation of Human Adipose Derived Stem Cells on Acellular Scaffold

**DOI:** 10.34172/apb.2020.075

**Published:** 2020-08-09

**Authors:** Hossein Taghavi, Jafar Soleimani Rad, Ahmad Mehdipour, Ahad Ferdosi Khosroshahi, Raziyeh Kheirjou, Milad Hasanpour, Leila Roshangar

**Affiliations:** ^1^Stem Cell Research Center, Tabriz University of Medical Sciences, Tabriz, Iran.; ^2^Department of Anatomical Sciences, Faculty of Medicine, Tabriz University of Medical Sciences, Tabriz, Iran.; ^3^Department of Tissue Engineering, Faculty of Medicine, Tabriz University of Medical Sciences, Tabriz, Iran.; ^4^Department of Biochemistry, Faculty of Medicine, Tabriz University of Medical Science, Tabriz, Iran.

**Keywords:** Adipose derived stem cells, Proliferation, Small intestine submocusa, Acellular scaffold

## Abstract

***Purpose:*** Acellular scaffold extracted from extracellular matrix (ECM) have been used for constructive and regenerative medicine. Adipose derived stem cells (ADSCs) can enhance the vascularization capacity of scaffolds. High mobility group box 1 (HMGB1) and stromal derived factor1 (SDF1) are considered as two important factors in vascularization and immunologic system. In this study, the effect of mineral pitch on the proliferation of human ADSCs was evaluated. In addition to HMGB1 and SDF1, factors expression in acellular scaffold was also assessed.

***Methods:*** To determine acellular scaffold morphology and the degree of decellularization, hematoxylin & eosin (H&amp;E), 6-diamidino-2-phenylindole (DAPI), and Masson’s trichrome staining were applied. The scaffolds were treated with mineral pitch. Also, ADSCs were seeded on the scaffolds, and adhesion of the cells to the scaffolds were assessed using field emission scanning electron microscopy (FE-SEM). In addition, the efficiency of mineral pitch to induce the proliferation of ADSCs on the scaffolds was evaluated using 3-(4, 5-dimethylthiazol-2-yl)-2, 5-diphenyl tetrazolium bromide (MTT) assay. To measure HMGB1 and SDF1 mRNA expression, real-time polymerase chain reactions (RT-PCR) was used.

***Results:*** FE-SEM showed that decellularized matrix possesses similar matrix morphology with a randomly oriented fibrillar structure and interconnecting pores. No toxicity was observed in all treatments, and cell proliferation were supported in scaffolds. The important point is that, the proliferation capacity of ADSCs on Mineral pitch loaded scaffolds significantly increased after 48 h incubation time compared to the unloaded scaffold (P<0.001).

***Conclusion:*** The results of this study suggest that mineral pitch has potentials to accelerate proliferation of ADSCs on the acellular scaffolds.

## Introduction

Tissue engineering is the science of creating suitable substitutes for body organs and tissues.^1,2^ The three main bases of tissue engineering are scaffolds, cells, and growth factors to overcome limitation in organ transplant.^3,4^ Scaffolds can form an integral part of tissue engineering as an extra cellular matrix for cell growth and proliferation. Decellularized biological scaffolds have advantages over synthetic scaffolds, including being natural, being easily available, and economical.^5-7^ They can be extracted from various tissues including the bladder,^8^ small intestine,^9^ liver^10^ and tracheal tissues.^11^

Stem cells are also highly differentiable cells widely used in tissue engineering. They can be extracted from different sources such as bone marrow, adipose tissue, and derma.^12^ Adipose derived stem cells (ADSCs) have shown to possess considerable proliferation potential as well as secretion of cytokines, growth factors, and angiogenic factors.^13,14^ In addition, ADSCs are able to interact with acellular scaffolds.^15^ However, researchers have made no coordinating efforts to improve the growth and proliferation of these cells on acellular scaffolds. Therefore, natural materials such as mineral pitch, plants, and their derivatives could be examined for this purpose.

Mineral pitch is a natural substance, which is generally found at heights with a color ranging from Brown to black. This substance is formed on some certain plant species due to the activity of microorganisms.^16,17^ Also, it contains sulphur, magnesium, nitrogen, polysaccharide, and oxygen.

Mineral pitch can be found in two forms as water-soluble and fat-soluble. For topical application, it is dissolved in sweltering water and then massaged on to the affected place such as a wound or a swollen joint.^18^ In traditional medicine, mineral pitch was used to treat bone fracture and wounds.^19^

High mobility group box 1 (HMGB1) is a gene associated with cell damage, which is involved in intracellular homeostasis. Accordingly, it induces inflammatory responses by exciting the cytokines and chemokines. This gene was released by macrophages as a result of necrosis. Also, as Recent research suggested, this type of gene remains in acellular scaffolds and stimulate the immune system.^20,21^ However, the expression of this gene in acellular scaffolds caused by loading stem cells or other substances, has not been investigated yet.

Stromal derived factor1 (SDF1) is an important regulatory factor contributing to wound healing through angiogenesis.^22^ A higher expression of SDF1 gene has been shown during healing process of human wounds.^23^ According to a recent study, the transmission and concentration of stem cells in target tissues are resulted from the combination of this factor with biologic scaffolds.^24^ Also, another study proved the effect of this factor on angiogenesis. This gene was originally extracted from the stromal cells on mice bone marrow, while it is expressed in the stromal cells of some tissues including the small intestine tissue.^25^ However, no study has explored the expression of this gene in acellular scaffolds under the effect of cells or other materials.

Acellular scaffolds provide a proper environment for cell growth; however, the use of materials promoting cell growth can be effective. The substance that we used in this study was mineral pitch; therefore, we examined its effect by adding an effective dose to the acellular scaffold.

Furthermore, analyzing the expression of HMGB1 and SDF1 genes in acellular scaffolds under the effect of mineral pitch were evaluated.

## Materials and Methods

### Preparation of extracellular matrix from sheep small intestine

Preparation of extracellular matrix (ECM) from small Intestinal sub mucosa (SIS) has been described earlier.^26,27^ In this study, different methods were used in order to decellularization. Briefly fresh sheep jejunum was harvested from a local slaughterhouse ( weighting 100-200 lbs), and was then washed by water before being conveyed in phosphate-buffer saline (PBS: Sigma, USA) Containing 100 mg/mL streptomycin and 100U/mL penicillin (Sigma, USA). Specimens were engrossed for 48 hours in saline before being longitudinally cut into 12 cm fragments. Also, tissue mucosa was mechanically removed. The external tunica muscularis and serosa layers were removed using a very sharp and small lancet. Chemical part of decellularization was done using sodium dodecyl sulfate 0.05% (SDS: Sigma, USA). The mechanical Part of decellularization was accomplished using agitation (100 rpm) and temperature (37°C). A cleaning step was performed using distilled water for 30 minutes, following reagent treatment and carrying out the procedure. Staining techniques were applied for confirmation of decellularization process.

### Histological assessment 

To characterize decellularization rate, decellularized and untreated sections were fixed in 10% neutral buffered formalin (Sigma, USA). For dehydration, they were passed through increasing concentrations of ethanol (50%, 70%, 90%,100%) for 35 minutes, immersed twice in xylene for 35 minutes, and were then stained using hematoxylin & eosin (H&E) to determine the rate of decellularization, and after that the number of removed nuclei was calculated. For further confirmation of remaining nuclei in decellularized scaffolds, 6-diamidino-2-phenylindole (DAPI) staining (Sigma-Aldrich, USA) was used and examined using fluorescence microscope (OPTIKA- XDS-3FL4, Italy, X10 and 40) by applying applicable filters. To evaluate the collagen status in the decellularized tissue, the section from both control and treated groups was stained with Masson’s trichrome.

### Mineral pitch preparation

Mineral pitch was purchased from the local market in Tabriz city. Effective mineral pitch concentration was determined as described earlier.^28^ Since mineral pitch is water-soluble, it was completely solved in low glucose Dulbecco’s modified eagle medium (DMEM), and filtered through 0.22 μm syringe filter for sterilization, and was then loaded on scaffolds whenever needed

### Field emission scanning electron microscopy (FE-SEM)

FE-SEM was used to examine the attachment of the ADSCS onto the ADSCs-loaded scaffolds.^29^ ADSCs was used at the density of 40 000 cell onto specimens. After sterilization using ultraviolet (UV) light, cells with mineral pitch and also without it, were seeded onto the decellularized scaffolds, and were then incubated at 37°C. The culture media was removed, and samples were washed using PBS and were then incubated in 2.5% glutaraldehyde for 45min and at 24°C. Glutaraldehyde was then removed and specimens were washed with PBS. Dehydration was carried out using a series of increasing concentration of ethanol (30%, 50%, 70, 80%, 90%, and 100%), 15 minutes for each concentration. Samples were finally dried, covered by one nm thickness golden layer, and were then studied using an FE-SEM device (PHILIPS- XL 30, USA).

### Mechanical test

In bioscaffolds, mechanical properties are considered as one of the essential factors. Scaffolds must meet the proper mechanical characteristics along with its biological performance. Experimental Tensile tests were performed according to ASTM638 standard over a cellular scaffold for three times. Samples were produced with exact dimensions, and GOTECH (AI-7000M-Taiwan) machine was used for experimental tests at constant tensile velocity of 2 mm/min.

### Cell viability assay by MTT

Proliferation and the cell viability were assessed using 3-(4, 5-dimethylthiazol-2-yl)-2, 5-diphenyl tetrazolium bromide (MTT; Sigma, USA) assay method, in which the yellow tetrazolium MTT was reduced by mitochondrial enzyme named as succinate dehydrogenase to produce intracellular bluish purple formazan, which is measurable.^30,31^ The control group included recellularized scaffolds without mineral pitch substance, and treatment group included recellularized scaffolds with mineral pitch substance. MTT assay was performed in 24-well cell culture plate to assist manipulation. Also, ADSCs was seeded at density of 10 000 cell into each well, and then the dishes were incubated at 37°C. Medium was removed 24 and 48 hours after cells were seeded, and 200 mL MTT solution (0.5 mg/mL) was added to each sample. By passing 3 hours from incubation at 37°C, MTT solution was removed, and 400 mL Dimethyl sulfoxide (DMSO) was added, then pipetting was used to destroy the cells and also to extract formazan crystals. One hundred microliters of the supernatant obtained from treated and untreated groups were transported into 96-well cell culture plates, and the absorbance of the formazan product was measured at 570 nm using an Elisa reader (Elx 808 Biotech, USA).

### Real-time polymerase chain reactions (RT-PCR)

To estimate HMGB1 and SDF1 mRNA in the control and treated specimens, RNA expression was determined using RT-PCR test. The primers used in the RT_PCR are reported in Table 1. Glyceraldehyde-3-phosphate dehydrogenase (*GAPDH* ) was used as the internal control gene to standardize the consequences.

**Table 1:** Primer process for real-time reverse transcription-polymerase chain reaction

**Gene**	**Sequence( 5’_3’)**
HMGB1	F: CCCAAAAGCGTGAGCTTAAAAT
	R: AGTCTCGTTTCCTGAGCAGTCC
SDF1	F: TCAGCCTGAGCTACAGATGCCCA
	R: GCCGGGCTACAATCTGAAGGGC
GAPDH	F: CGGGGTCCCAGCTTAGGTTC
	R: GCCCAATACGGCCAAATCCG

RNAs were extracted using RNeasy Micro kit (Denazist, Iran). In addition, primers were designed and RT-PCR was conducted to analyze the gene expressions using SYBR green technology.

### Statistical analysis

Statistical analysis was performed using *t* test and done with GraphPad Prism statistic software version 8. Results were expressed as mean ± SD, and *P* <0.05 was considered as statistically significant.

## Results

### Histological findings

**Figure 1:**
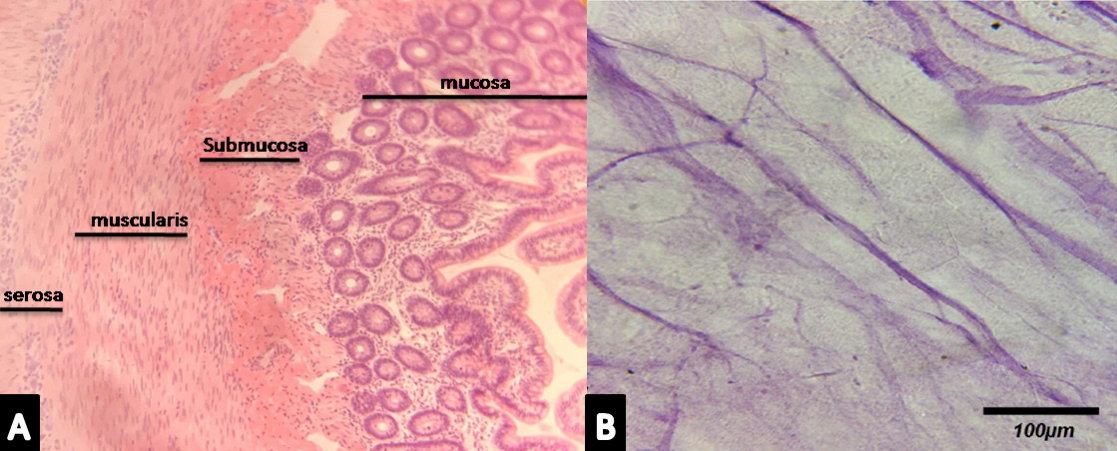
A) Photomicrograph of intestine section showing mucosa and submucosa, cell nuclei as blue dots in submucousal layer, H&E staining, 40X. B) A section of decellularized SIS showing no nuclei, H&E staining, 40X.

Evaluation of decellularization rate, using H&E, showed that almost 95% of cellular nuclei were eliminated. However, some remnant of cell nuclei appeared to be present in decellularized tissue as shown in Figure 1.

**Figure 2:**
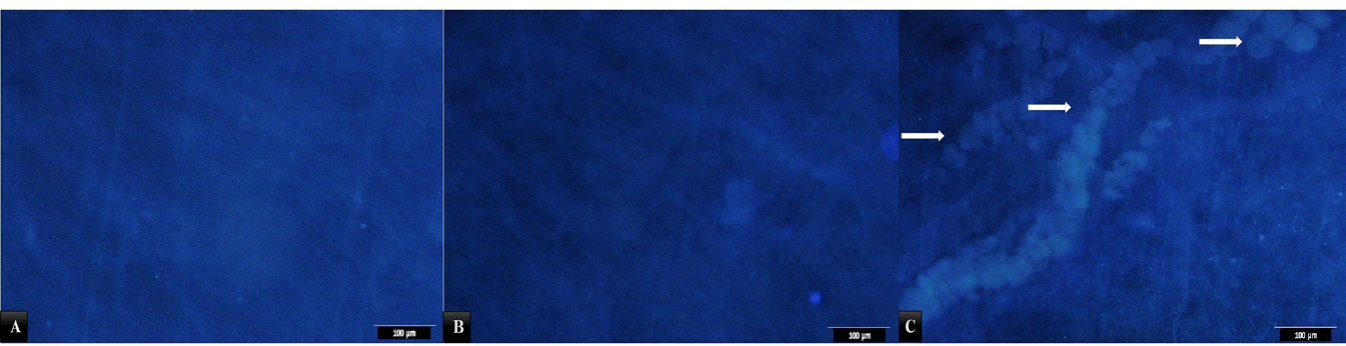
A) Photomicrograph of submucosal section from control group stained with DAPI. Cell nuclei in blue color. B) A section of acellular SIS stained with DAPI. Note disappearance of nuclei. C) Normal tissue with cells. The arrows show cells of the scaffold.

DAPI staining as a specific staining for nuclei confirmed the findings with H&E as indicated in Figure 2. However, some remnant of cell nuclei appeared to be present in decellularized tissue.

**Figure 3:**
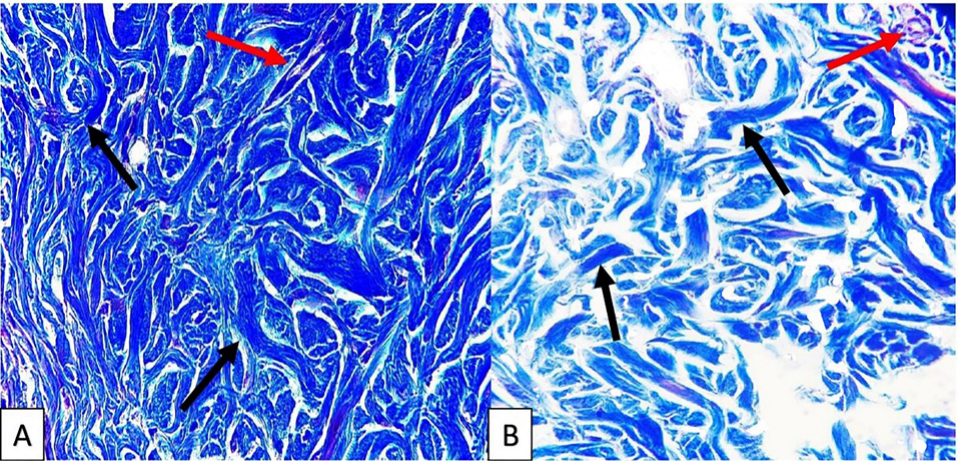
Photomicrograph of sections from cellular (A) and decellular (B) SIS that showing no difference in collagen content, the red arrows show blood vessels and the yellow arrows show collagen of the tissue, Masson’s trichrome staining, 40X.

Results of Masson’s trichrome staining showed that collagen content of cellular and decellular SIS are similar, as indicated in Figure 3.

### FE-SEM findings

**Figure 4:**
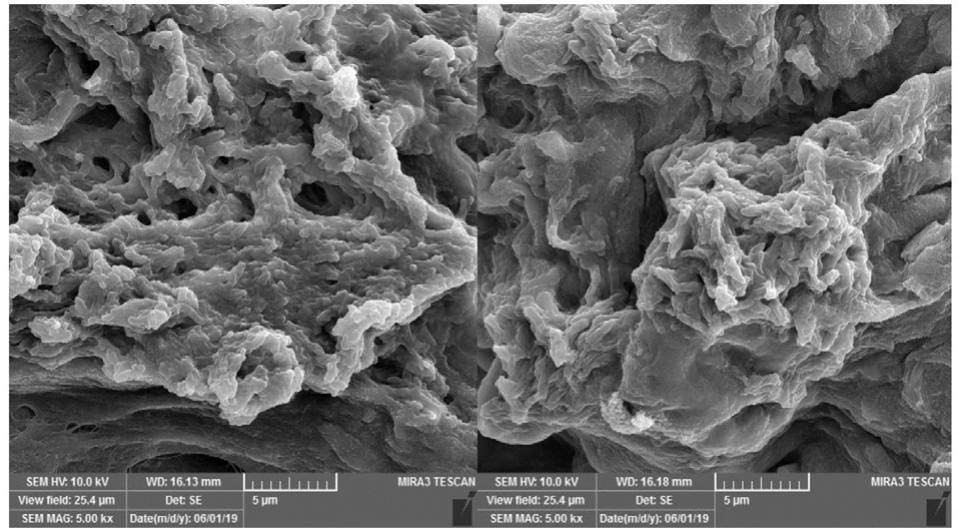
FE-SEM images of cellular showing that fibrillar structures and interconnecting pores, are similar in control and mineral pitch-treated groups.

**Figure 5:**
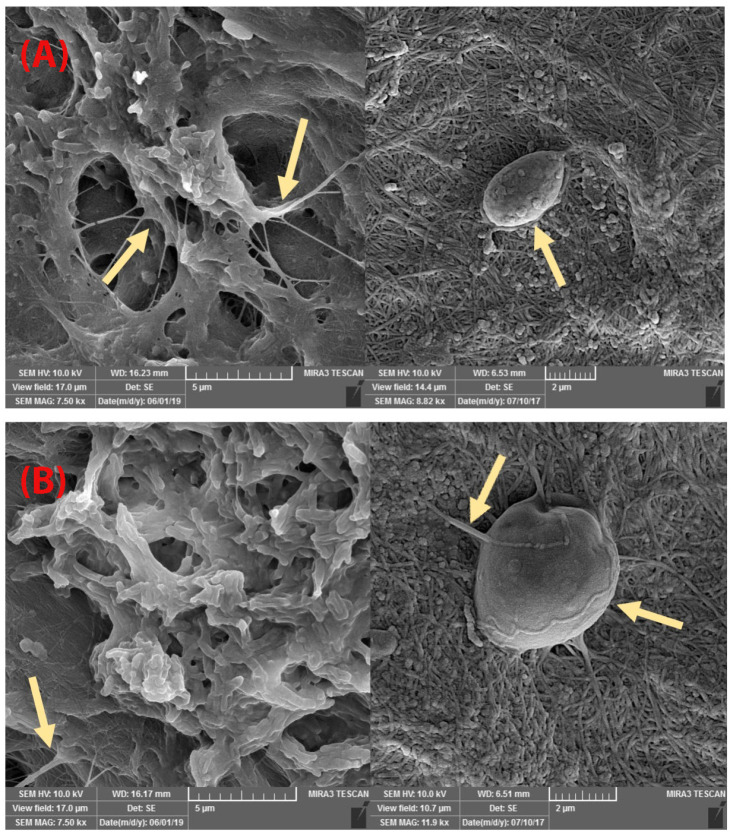
FE-SEM images of seeded ADSCs on scaffolds. A) Recellular scaffold without mineral pitch (control group). B) Recellular scaffold with mineral pitch (treated group). The arrows show the cells. These images similarity of cellular adherence to scaffolds in both groups.

FE-SEM images showed that the mineral pitch loaded and control group matrices possesses similar matrix morphology with a randomly oriented interconnecting pores and fibrillar structure as shown in Figure 4. Also, SEM-images revealed that decellularization removed all the cellular content. Examination of mineral pitch treated/control acellular scaffolds with SEM, by passing 24 hours from seeding of them with ADSCs showed several adhered cells in both groups, as presented in Figure 5.

### Ultimate tensile strength

According to the tensile test, acellular tensile strength was 139±4 kPa. Results indicated that acellular scaffold had proper tensile strength.

### Cell viability 

**Figure 6:**
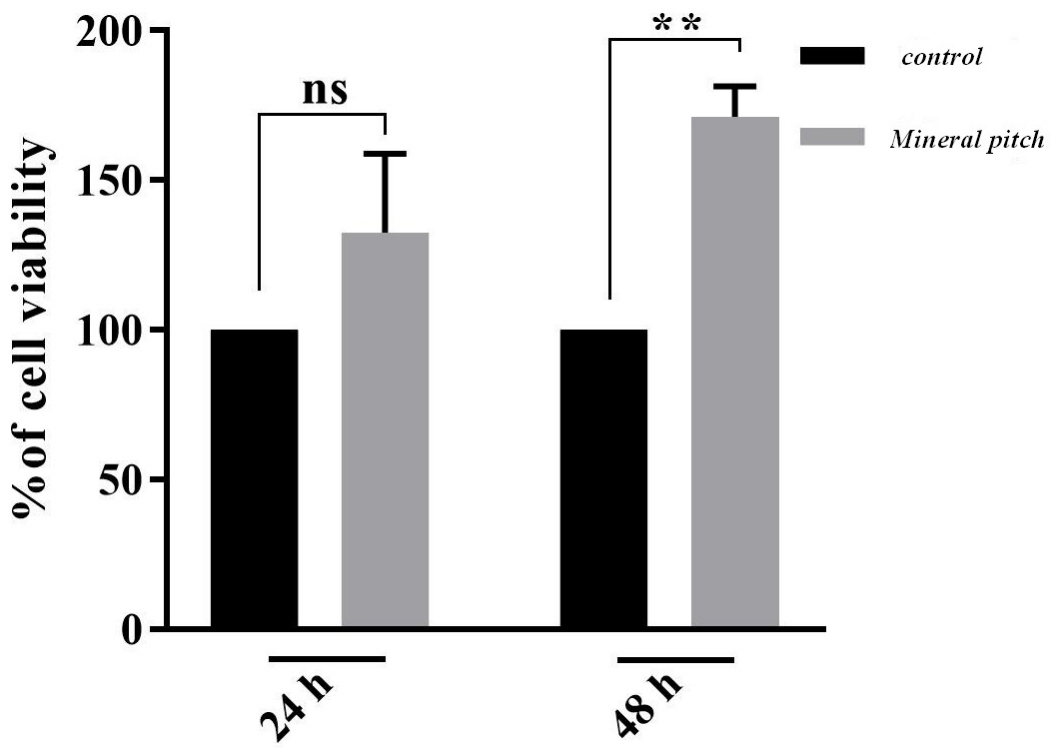
MTT assay diagram in 24 and 48 hours after seeding of ADSCs on treated scaffolds in comparison with control group (*P* <0.001).

The MTT assay analysis revealed that the calculated optical density values that were measured over 24 hours, were not significantly different between the group that was treated with 1000 μg/mL of mineral pitch and control group. However, the viability of ADSCs in mineral pitch-loaded scaffold significantly increased after 48 hours, compared to control group as shown in Figure 6.

### Real-time polymerase chain reactions (RT-PCR)

**Figure 7:**
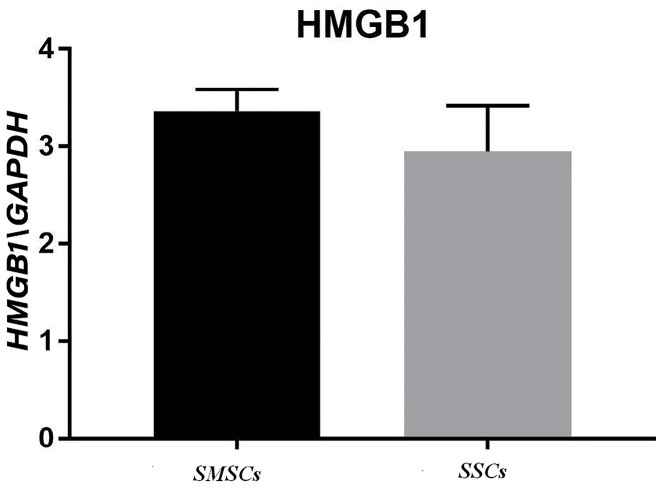
HMGB1 gene expression in recellularized scaffold with mineral pitch and stem cells (SMSCs) and recellularized scaffold without mineral pitch (SSCs) that shows significant increase in treated group with mineral pitch (*P* <0.01).

**Figure 8:**
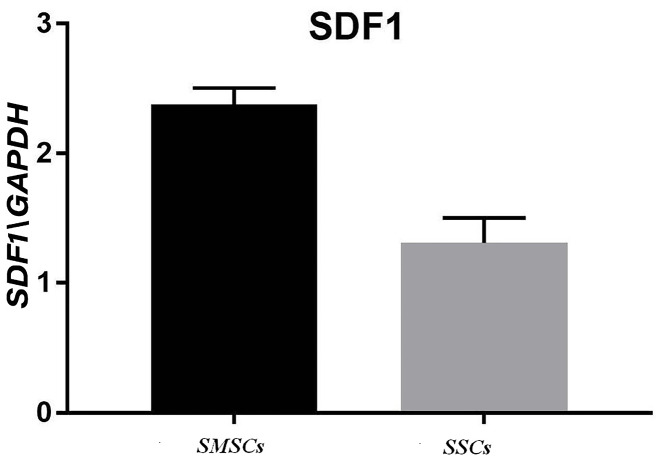
SDF1 gene expression in recellularized scaffold with mineral pitch and stem cells (SMSCs) and recellularized scaffold without mineral pitch (SSCs) that shows significant increase in treated group with mineral pitch (*P* <0.0001).

A significant rise was perceived in HMGB1 mRNA in the acellular scaffolds seeded with ADSCs during treating with mineral pitch compared to the control group (P<0.01; Figure 7). Moreover, significant differences were observed in SDF1 gene expression between treated and untreated groups (P<0.0001; Figure 8).

## Discussion

In our study, acellular scaffold was successfully synthesized, and decellularization was confirmed by histological staining. SEM examined scaffolds and provided additional evidence for removal of cellular content and for more confirming an almost complete decellularization. The scaffold promoted cellular adhesion and proliferation, which means that it could be effective for wound healing and tissue regeneration.

The result of Scanning electron microscopy showed that treating of acellular scaffold with mineral pitch do not change adhesion ability of the acellular scaffold.

MTT assay revealed that cellular viability in mineral pitch-treated group increased, in comparison with control group

Regarding the fact that, in 24 hours interval, the optical density was significantly higher in treated group than control group; therefore, it can be concluded that cellular proliferation in treated group significantly increased compared to control group. This indicates that mineral pitch has the ability of promoting the proliferation potential of ADSCs and could mimic this function in *in vivo* . In agreement with our finding, Soleimani et al.^32^ have showed that mineral pitch is effective on cellular migration and proliferation in vitro. Migration and proliferation of cells in acellular scaffold requires a structural network support, angiogenesis, chemotaxis, and release of several growth factor.^33^

It is obvious that one of the important challenge in tissue regeneration is a network to provide ECM with the ability of exchanging and diffusion of oxygen, nutrient, biochemical, and waste.

Lack of such a network could result in cellular deterioration and tissue dysfunction followed by necrosis. Beside the ECM, proteins, glycoprotein, glycoside, and collagen, some of biological and regulatory factors play important role in relation to wound healing therapy and angiogenesis.^23^ One of the essential proteins for wound healing is SDF1. According to some studies, during healing process of wounds, expression of SDF1 gene has been reported.^24^ In addition, this factor has an induction effect on angiogenesis in ECM. HMGB1 is another protein that was expressed during wound healing and is involved along with induction of inflammatory response via chemotaxis, and is an important factor for cellular immune homostasis.^21,22^

In our study, it was shown that expression of both SDF1 and HMGB1 were higher in recellular scaffold treated with mineral pitch than in control group. Also, it was Indicated that mineral pitch stimulates production of these proteins by ADSCs.

The complex of functional and structural glycoproteins, proteoglycans, and protein must be retained in 3D structural of acellular scaffolds. This 3D structure provides a key network for wound healing. Positive roles of combing acellular dermal matrix with mesenchymal stem cell and fibroblast were demonstrated in several in vitro studies.^34^

In our study, evaluation of collagen content in decellularized submucosa, their structural appearance with SEM, and ADSCs attachment to acellular scaffold are in favor of the above-mentioned findings.

Several studies clearly reveled that mesenchymal stem cell, which is special ADSCs, promotes human dermal cells proliferation by direct cell-to-cell contact and also via paracrine effect. Indirect increase of collagen and ECM composition synthesis is another effect of loaded ADSCs to dermal healing.^35^

## Conclusion

Finding of the study indicate that treating of acellular scaffold with mineral pitch could promote cellular viability, proliferation, and synthesis of several factors involved wound healing. Therefore, it could be concluded that combining of acellular submucosa, ADSCs, and mineral pitch may effectively accelerate wound healing in vivo.

## Ethical Issues

The ethical committee of Tabriz University of Medical Science (No. IR.TBZMED.VCR.REC.1397.094) approved the study procedure.

## Conflict of Interest

Authors declare no conflict of interest in this study.

## Acknowledgments

This project is the result of a master’s degree proposal and the authors are thankful for stem cell research center of Tabriz University of medical science for support.
